# “Arm in arm with righteousness”

**DOI:** 10.1186/s13010-015-0024-y

**Published:** 2015-04-23

**Authors:** Iona Heath

**Affiliations:** Immediate Past President, Royal College of General Practitioners, 30 Euston Square, London, NW1 2FB UK

## Introduction

My title comes from Joseph Conrad who, in his 1913 novel *Chance*, wrote:

You know the power of words. We pass through periods dominated by this or that word - it may be development, or it may be competition, or education, or purity or efficiency or even sanctity. It is the word of the time. Well just then it was the word Thrift which was out in the streets walking arm in arm with righteousness, the inseparable companion and backer up of all such national catch-words, looking everybody in the eye as it were [1].

Today, the word is quality, out in the streets walking arm in arm with righteousness, with all the self-satisfaction that implies. The fundamental problem with all such national catch-words is that they all too seldom have real substance and all too often become slogans used in the exercise of power.

The challenge is that there will never be authentic quality within healthcare unless the word explicitly accommodates the truth that a human being is simultaneously both a subject and an object. Within a consultation both doctor and patient need to oscillate between perceiving the body as an object and as a subject. When the body is perceived as an object, the gaze of biomedical science sees only what the particular patient has in common with other patients: the perspective is normative and monological. When the body is perceived as a subject, we see what is unique about this person: their life context, its story and the meanings that adhere to both. The perspective is dialogical and intersubjective involving two unique subjects: the patient and the doctor. Issues of quality need to be addressed within both of these perspectives. Yet, to date, quality in relation to the human being as an object has predominated because this is infinitely the easier option as it is possible to create a normative standard that is able to completely ignore the difficult issues of subjectivity [2].

### A stone or a bird

Systems engineer Paul Plsek compares throwing a stone with throwing a live bird. The trajectory of a stone can be calculated precisely using the laws of mechanics, and it is possible to ensure that the stone reaches a specified target [3]. However, it is absolutely not possible to predict the outcome of throwing the live bird, even though, in truth, the same laws of physics govern the bird’s motion through the air. As Plsek points out, one solution would be to tie the bird’s wings, weight it with a rock and then throw it. This will make its trajectory nearly as predictable as that of the stone, but in the process the capability of the bird is completely destroyed. This seems very close to what happens when we try to measure the quality of healthcare using measures that ignore the presence of human subjects either as patients or as healthcare professionals.

The anthropologist Clifford Geertz touches on the same contrast -

The contrast … is … between those who believe that the task of the human sciences … is to discover facts, set them into propositional structures, deduce laws, predict outcomes, and rationally manage social life, and those who believe that the aim of those sciences … is to clarify what on earth is going on among various people at various times and draw some conclusions about constraints, causes, hopes, and possibilities - the practicalities of life [4].

And he argues the need to hold these polarities in the sort of constructive balance that seems so far to have eluded us in the assessment of quality in healthcare.

The flight into scientism, or, on the other side, into subjectivism, is but a sign that the tension cannot any longer be borne, that nerve has failed and a choice has been made to suppress one’s humanity or one’s rationality. These are the pathologies of science, not its norm.

So far, in the pursuit of quality, we have exploited rationality at the expense of humanity [5].

The great German philosopher Hans Georg Gadamer writes:

- the progress of technology encounters an unprepared humanity. It vacillates between the extremes of an affect-laden opposition to rational innovation and a no less affect-laden craving to ‘rationalize’ all forms and sectors of life, a development which more and more acquires the form of a panic flight from freedom [6].

Today, much of the assessment of quality in healthcare feels like this panic flight from freedom and we are more and more subjected to ‘the tyranny of what can be measured’ [7]: the endless ticking of boxes and completion of forms and entertaining of teams of inspection and regulation [8].

### Surveillance

Computers allow the processing of unprecedented amounts of data and they are driving an obsession with measurement and it is being used in a normative and coercive way to define and demonise “deviant” behaviour whether among doctors or patients.

Writing about the development and use of psychological tests, Tor-Johan Ekeland describes such measurements as rendering:

- individuals into knowledge as objects of a hierarchical and normative gaze. The individuality is no longer unique and beyond knowledge, but can be known, mapped, calibrated, evaluated, quantified, predicted and managed. They become techniques for the disciplining of human difference [9].

Biological variation has been appropriated to the causes of commercial profit and of lifestyle and political conformity, and normative quality assurance that focuses on the body as an object is part of this. In an increasingly individualistic and consumerist society, people are encouraged to seek “the best” both now and in the future. Nothing can be left to chance, every risk must be minimised and the emphasis is on control. Physicians may not be driving this but they are certainly colluding.

The culture of conformity pays lip service to autonomy and choice but it is clear that the individual is only really free to make the choice which is approved by the state [10]. It is assumed that once the “healthy choice” is pointed out, everyone will select it and no account is taken of the very differing circumstances and aspirations of different people’s lives.

Across the whole of medicine, we are witnessing an extraordinary process within which the definition of disease is shifting away from patients’ subjective experience of symptoms, distress and suffering and towards the numbers of biometric measurement.

Longevity has become the preeminent outcome measure and the length of life has become more important than how that life is lived. Yet, as critic Christopher Ricks puts it, although -

Most people most of the time want to live forever. … most people some of the time, and some people most of the time, do not want to live forever [11].

In 1941 Rebecca West wrote -

- only a part of us is sane: only part of us loves pleasure and the longer day of happiness, wants to live to our nineties and die in peace, in a house that we built, that shall shelter those that come after us. The other half of us is nearly mad. It prefers the disagreeable to the agreeable, loves pain and its darker night despair, and wants to die in a catastrophe that will set back life to its beginnings and leave nothing of our house save its blackened foundations [12].

If medicine and health care systems, and their associated quality measures, can offer no understanding of this other half, they will fail – drug dependence, alcoholism, mental illness, suicide, violence and much ordinary illness will elicit no response.

The contemporary marginalisation of the human subject affects doctors and other healthcare professionals almost as much as patients. Both have been reduced to interchangeable units within a healthcare industry: the one as a unit of healthcare need, the other of healthcare provision. And, as patients have been reduced to these interchangeable units of health need, their access to the system has been systematically prioritised over the need to sustain a relationship with a known and trusted professional, with another human subject.

### Quality and outcomes framework

In UK general practice, the Quality and Outcomes Framework has exemplified the growing ascendancy of the body as object over the body as subject. It has provoked reactions ranging from enthusiasm to dismay but because of the substantial financial incentive the points have been collected assiduously. The stated ambition is that the quality criteria should be evidence based but most evidence is derived from the study of highly selected populations with a single condition and from only a segment of the age range. Nonetheless, the QOF criteria are applied across the whole age range and to those with complex co-morbidity thereby extrapolating the evidence well beyond its initial range. This systematic extrapolation across age, gender, ethnicity, and social, cultural and economic context should be a serious cause for concern. To date, the vast majority of the clinical measures have been of process rather than of outcomes relevant to patients and a disturbingly large number of the measures assess the adherence to pharmaceutical treatments.

The QOF purports to measure clinical quality at the level of the individual, or rather at the level of a standardised normative individual of its own creation, and organisational quality at the level of the primary care practice. Take the case of a single patient with hypertension. However, she also has bronchiectasis after many years of smoking. She has just been diagnosed with cancer of the oesophagus. She has a child with severe learning difficulties and she is fearful, not only for herself but also about what will happen to her child. She is married and the relationship is difficult. Her sister has lung cancer and is already very ill. The sister’s children have problems and children of their own and this is only the beginning of a story which becomes richer and richer the more it is told. It is a story with multiple components each of which interacts with the others unpredictably. Each of the components has a history which affects the interaction and each has the capacity to affect the patient’s blood pressure and to support or undermine the treatment that is prescribed for her. To what extent does the achievement of a blood pressure of less than 150/90 assess the quality of this patient’s care?

Don Berwick – perceptive as ever, notes:

Individuals involved in day-to-day improvement work fear that if “evidence” is too narrowly defined and the approach to gathering evidence too severely constrained, progress may be the victim [13].

The messy reality of primary care is that most patients have multiple, interacting and compounding problems – physical, psychological and social. However, most scientific evidence upon which disease-specific quality measures are based, explicitly excludes people with co-morbid conditions. When such patients are seen by a number of specialist physicians, each of which has expertise in a single condition, the sum of the advice can be both conflicting and excessively burdensome to the patient. The whole patient is more than the sum of the parts. The key tasks of primary health care are the integration of care so that it becomes both possible and coherent, personalising care to the particular circumstances and capacities of the individual patient, explicitly acknowledging the human subject, and prioritising the various problems so that the burden on the patient is minimised [14]. We have no metric for measuring these qualities. And if we could create one, would it make the task any easier [15]?

In contemporary healthcare, numbers have, to a very large extent, taken the place of words; yet, description is so much more capacious than measurement. Perhaps we should be assessing quality much more through story and description and much less through number and reductive processes of measurement. The problem, of course, is that the assessment of words necessarily involves human judgment and cannot be done by a computer. Yet this is perhaps the one attribute of words that make them so peculiarly appropriate for judging quality within healthcare.

### The predicament of professionals

As austerity and socioeconomic polarisation make more and more people sick, and as the quality machine demands more and more reporting, the pressure on general practitioners and other healthcare professionals is becoming intense. And there is no time for reflection and learning, no time for improvement, more failure, and so, yet more inspection and regulation is imposed: this particular vicious circle has become very familiar and seems to be wound tighter every week.

Clinical medicine provides the arena within which the subjective experience of illness and suffering is brought into contact with the classifications of biomedical science. The evidence on which biomedical science is based has been derived from the analysis of data from populations grouped together by what they have in common: difference is systematically excluded or ignored. Huge advances in understanding and efficacy have been made through these techniques. The ineradicable problem is that data collected from populations can tell us nothing about what will happen to any particular individual. The application of biomedical evidence to the care of an individual will always require the exercise of judgment on the part of both clinician and patient. What is the appropriate label to use for this person in this particular situation and which treatment is both acceptable and the most likely to help rather than harm? In primary health care, these judgments are often made in the context of an ongoing relationship between patient and clinician and the nature of the relationship can affect the outcome for either good or ill. Hence, the quality of primary healthcare is crucially dependent on two phenomena, judgment and human relationships, and for neither of which is there any recognised metric of assessment.

In 1956, the writer George Ewart Evans published his masterpiece of oral history which he called ‘Ask the Fellows who Cut the Hay’ [16]. It would be more than timely if those in power in this country could be persuaded to reflect on this title. There is a pervasive and disturbing lack of knowledge of the daily experience of working at the frontline of public service, let alone any valuing of or respect for that experience. This applies to teachers, social workers, civil servants, police, nurses, doctors and many more. It is undoubtedly true for those working in general practice and primary care which is particularly sad as we see, every day, the effects of structural violence and social injustice working themselves out in premature illness and disease and in blighted and shortened lives.

The fellows who cut they hay in the health service would, I think, agree with Annmarie Mol -

Our theoretical frameworks seem to be too exclusively adapted to the task of ‘criticism’. They unmask. They tend not to explore or build ideals but to undermine them [17].

### Conclusion

In his essay on Virgil published in 1944, TS Eliot wrote

In our age, when men seem more than ever prone to confuse wisdom with knowledge, and knowledge with information, and to try to solve problems of life in terms of engineering, there is coming into existence a new kind of provincialism which perhaps deserves a new name. It is a provincialism, not of space, but of time; one for which history is merely the chronicle of human devices which have served their turn and been scrapped, one for which the world is the property solely of the living, a property in which the dead hold no shares [18].

This underlines the disturbing tendency within medicine to live outside history: to mock the mistakes of previous generations while assuming the enduring rightness of our own priorities and procedures. Modern quality machinery involves insufficient doubt and it has become difficult to question the means because the end of ‘quality’ is so obviously worthy. Nonetheless, many of the means are damaging not least because they are so unidimensional and propagate an intensely normative and objectifying view of what it means to be healthy and of what human life and healthcare should be [19].

I accept that, as in the consultation, when we try to assess the quality of health care we need to hold in balance the object and the subject. The problem is that the object side of the balance is so much easier to assess that it tends to overwhelm the subject – and the human subject is in so many aspects unknowable that attempts to bolster the subjective assessment of quality may prove destructive of aspects of practice which, at their best, are ephemeral, ineffable and unmeasurable.

So let me finish by going back to Conrad: what would the world look like if we went back to thrift and put thrift back arm in arm with righteousness? It would look very very different and perhaps be none the worse for that.

We would at least have revitalised

- an acute sense of waste as a moral and political issue [20].

At the moment we waste effort, money and time, collecting data and pursuing quality targets, so that we have less time to listen and we risk losing sight of the suffering human subject. And we risk destroying quality in our attempt to measure it. It is time to untie Plsek’s birds.
